# Fluorescence Fluctuations and Equivalence Classes of *Ca* ^2+^ Imaging Experiments

**DOI:** 10.1371/journal.pone.0095860

**Published:** 2014-04-28

**Authors:** Estefanía Piegari, Lucía Lopez, Emiliano Perez Ipiña, Silvina Ponce Dawson

**Affiliations:** Departamento de Física and IFIBA (CONICET), FCEyN-UBA, Ciudad Universitaria, Pabellón I, Buenos Aires, Argentina; Dalhousie University, Canada

## Abstract

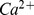
 release into the cytosol through inositol 1,4,5-trisphosphate receptors (IP_3_Rs) plays a relevant role in numerous physiological processes. IP_3_R-mediated 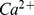 signals involve 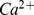-induced 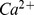-release (CICR) whereby 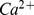 release through one open IP_3_R induces the opening of other channels. IP_3_Rs are apparently organized in clusters. The signals can remain localized (i.e., 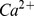 puffs) if CICR is limited to one cluster or become waves that propagate between clusters. 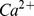 puffs are the building blocks of 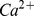 waves. Thus, there is great interest in determining puff properties, especially in view of the current controversy on the spatial distribution of activatable IP_3_Rs. 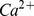 puffs have been observed in intact cells with optical techniques proving that they are intrinsically stochastic. Obtaining a correct picture of their dynamics then entails being able to detect the whole range of puff sizes. 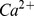 puffs are observed using visible single-wavelength 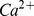 dyes, slow exogenous buffers (e.g., EGTA) to disrupt inter-cluster CICR and UV-photolyzable caged IP_3_. Single-wavelength dyes increase their fluorescence upon calcium binding producing images that are strongly dependent on their kinetic, transport and photophysical properties. Determining the artifacts that the imaging setting introduces is particularly relevant when trying to analyze the smallest 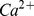 signals. In this paper we introduce a method to estimate the expected signal-to-noise ratio of 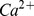 imaging experiments that use single-wavelength dyes. The method is based on the Number and Brightness technique. It involves the performance of a series of experiments and their subsequent analysis in terms of a fluorescence fluctuation model with which the model parameters are quantified. Using the model, the expected signal-to-noise ratio is then computed. Equivalence classes between different experimental conditions that produce images with similar signal-to-noise ratios can then be established. The method may also be used to estimate the smallest signals that can reliably be observed with each setting.

## Introduction

Calcium signals are ubiquitous [1]. Their versatility relies on the variety of spatio-temporal behaviors that the intracellular calcium concentration can display. 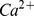
release into the cytosol through inositol 1,4,5-trisphosphate receptors (IP_3_Rs) is a key component of the 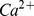
signaling toolkit [2]. Optical techniques and 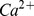 fluorescent dyes provide a relatively non-invasive means to study the dynamics of intracellular 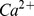 signals [3], [4], in particular, those that are IP_3_R-mediated. These observations have revealed a wide variety of signals that go from those that remain spatially localized to those that propagate throughout the cell [5]. The observations, however, are indirect. Most 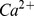 dyes change their spectral properties upon 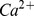 binding. Thus, the observed fluorescence is related to the 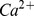-bound dye rather than the free 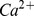
concentration. Having reliable estimates of the dye physical properties is then necessary to quantitate the underlying free 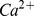
distribution [6]. IP_3_R-mediated 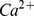
signals are observed using single-wavelength dyes that are excited with visible light and caged IP_3_ that is photolyzed with UV illumination to evoke the signals [7]. Differently from ratiometric dyes, single-wavelength dyes do not allow for a direct measurement of the 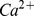 concentration [8], [9]. Fluorescence variations during 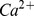
signals are then presented as ratios with respect to basal fluorescence (prior to signal evocation) when using single-wavelength dyes. Namely, they are shown in terms of the ratio: 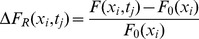
(1)where 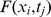 is the fluorescence at the pixel identified by the position 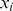 and time 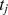 and 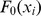 is the fluorescence at spatial point, 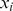, averaged over time prior to signal evocation. This minimizes the artifacts that spatial heterogeneities due to uneven dye distribution, specimen thickness or illumination intensity can introduce and allows a direct comparison across the image. The question naturally arises of whether this is enough to compare images performed under different experimental conditions. More specifically, how similarly a given underlying free 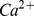
distribution is reflected in images that are obtained with different dyes and/or dye concentrations or with different experimental set-ups. This question is particularly relevant when the underlying dynamics is subject to Calcium Induced Calcium Release (CICR) (*i.e.*, when the 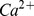
released through one open channel induces the opening of neighboring ones) since the dye or other substances introduced in the cells during the experiments can interfere with signal propagation.

IP_3_R-mediated 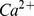
signals are subject to CICR. Namely, IP_3_Rs need to bind IP_3_ and 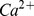
to become open [10]. In most cells IP_3_Rs are organized in clusters. Thus IP_3_R-mediated 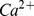
signals remain localized (e.g., puffs that involve the release through a few IP_3_Rs in a cluster) or global (e.g., waves) depending on CICR efficiency. Puffs are the building blocks of global signals and, as such, have been the subject of numerous studies [11]. Puffs are highly stochastic. On one hand, not all clusters contain the same number or spatial distribution of IP_3_Rs [3], [12]. On the other hand, the same cluster can give rise to puffs of different sizes depending on the number and spatial distribution of the activatable IP_3_Rs that there are at the beginning of the signal [13], two properties that change with time as IP_3_ binds/unbinds IP_3_Rs and IP_3_Rs enter or leave their inhibited state [14]. In order to obtain a good understanding of the dynamics of puffs it is then necessary to collect enough information so as to derive an accurate statistical description. The latter depends on the ability of the experimental set-up to detect most of the evoked events. The smallest ones, however, can easily go undetected depending on how large the change in fluorescence they produce is compared to spontaneous fluorescence fluctuations. Being able to detect the smallest possible events is particularly relevant in the case of IP_3_Rs since there is some controversy on what their actual spatial distribution is and how it can change with IP_3_ stimulation [15], [16]. In order to collect enough statistics on puffs, experiments are performed introducing a slow exogenous 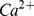
buffer (typically, EGTA) in the cell. In this way the evoked signals can be forced to remain local. The problem is that EGTA not only changes the dynamics of the signals. In principle, it can also modify the way that an underlying 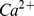 distribution is reported by the observed fluorescence. In this paper we introduce a method to estimate the signal-to-noise ratio of 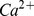
imaging experiments that use single-wavelength 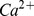
dyes and EGTA. The method relies on a model of fluctuations that can be quantitated by means of simple experiments. Once the model is quantified, numerically simulated 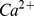
images with fluctuations that are in accordance with the used experimental setting can be generated. The quantified model can then be used to probe the limits of signal detectability of the experiments.

The method we introduce in this paper is based on the Number and Brightness (N&B) technique of [17]. N&B serves to separate the contributions of two sources of fluctuations that affect fluorescence images: variations in the number of molecules that contribute to the fluorescence at each pixel and variations in the number of detected photons. The N&B method has been applied mainly to analyze static images of samples containing fluorescent proteins and identify whether increments in the observed fluorescence are due to a change in the number of emitting molecules or in the brightness (i.e., the number of detected photons). To achieve this goal, images of the marked sample are obtained for different illumination powers. The mean and the variance of the fluorescence are then computed for each image. The analysis of how these quantities change with the illumination power allows a separation between number and brightness and between the mobile and the immobile fraction of the fluorescent molecules. In this way a pixel by pixel map of molecular number and aggregation can be obtained for the image. The aim of our method is to have a quantitative description of the distribution of fluorescence fluctuations when the emitters are single-wavelength 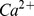
dyes in the presence of 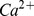. In this case, under stationary conditions (i.e., in the absence of 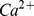
signals), there is an additional source of fluctuations with respect to those considered in the N&B method. Namely, single wavelength 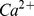
dyes emit in the same range of wavelengths if the dye molecules are bound or not to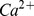, albeit, with a different quantum efficiency. 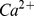 binding to the dye is a dynamic process. Thus, images obtained in the cytosol of cells containing one such dye, under stationary conditions, are also affected by variations in the fraction of 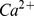-bound dye molecules that contribute to the fluorescence at each pixel. Furthermore, this fraction depends on the number of free 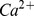 ions in the region of interest which is also a dynamic variable. Thus, the number of quantifiable parameters of the fluctuation model is larger in our method than in N&B and then requires more experiments. The purpose of our method, however, is more restricted than that of the N&B technique. We are not trying to establish a map of the number of dye molecules of 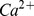 ions that are in the region that contribute to the fluorescence at each pixel. We are just interested in having a quantified version of the distribution of fluorescence fluctuations. With this type of information, assuming that the only change during the occurrence of 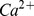
signals can be captured by a change in the probability that a dye molecule be bound to 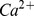, numerically simulated images of signals with realistic noise can be generated. As mentioned before, these images can then be used to determine the minimum 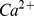
current that generates an observable change in fluorescence or, as done in this paper, to compute and compare the signal-to-noise ratio of different experimental settings. This last type application, in turn, can be used to optimize the dye and EGTA concentrations with which to observe the signals. Our method is particularly useful when experimentalists need to change the dye they have been using because it has been discontinued or because they want to try a new more efficient one. By means of our method experimentalists can compare the detectability properties of experiments performed with the old and with the new dye (e.g., Fluo-4 and Fluo-8) and also to estimate in which ways the dye concentration and/or illumination intensities could be modified to obtain comparable signal-to-noise ratios with both of them.

In this paper we present a general description of our method and then discuss it in more detail when applied to a particular example. For the example we obtain microscopy images, under stationary conditions (*i.e*., with no signal evocation), of *Xenopus lævis*'s oocytes injected with a single-wavelength 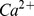 dye (either Fluo-4 or Rhod-2) and EGTA. We repeat the experiments varying the illumination power or the basal 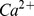 concentration. Analyzing the fluorescence fluctuations in terms of the fluctuation model of our method we quantify model parameters. We then use the quantified model to estimate the signal-to-noise ratio that can be expected from experiments performed using the combinations of dye and EGTA that are probed with the stationary experiments. We have chosen Fluo-4 and Rhod-2 for the example because they have different spectral (one is fluorescein and the other rhodamine-based) [9], [18] and kinetic properties (the on rate of Fluo-4 is supposed to be an order of magnitude larger than that of Rhod-2) [9], [18] and different affinities for 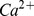
(according to the supplier, the dissociation constants are 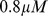
for Fluo-4 and 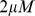 for Rhod-2). Having characterized the detection capabilities of two dyes with different spectral properties opens the possibility of using them simultaneously to study different aspects of 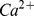 signals [19] or either one of them in combination with dyes that differentially enter the endoplasmic reticulum to monitor cytosolic and luminal calcium at the same time [20]. Fluo-4 in combination with EGTA has been the dye of choice to observe 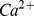 puffs in *Xenopus lævis* oocytes [11]. Rhod-2 has been less characterized for this type of applications. In particular, the choice of 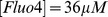
and 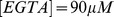
has proven to be adequate to observe 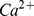 puffs [21]. As described later in this paper, using Rhod-2 and EGTA at these concentrations, puffs cannot be observed. Our method in fact estimates that the expected signal-to-noise ratio of these two experimental conditions differs by a factor of two. It places, on the other hand, in the same equivalence class in terms of the expected signal-to-noise ratio, experiments performed with 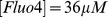
and 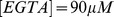 or 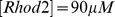
and 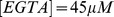, two conditions for which 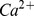
puffs are readily observable. In this example, an analysis of the noisy numerical images that can be generated using the quantified fluctuation model shed light on the reasons that underlie the differential ability of both dyes to detect 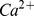
puffs at the same concentration. In particular, this exploration shows that using the ratio of the fluorescence variations during 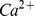
signals with respect to basal fluorescence is not enough to compare experiments performed at different dye and/or EGTA concentrations. We also show how the method can be used to quantify the variation of the signal-to-noise ratio if the dye and EGTA concentrations, the dye type or the illumination power are changed. This particular example then highlights the ability of our method as a tool for classification purposes and to compare or to improve the detectability conditions of different 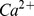
imaging experimental settings. The method, on the other hand, can be used to compare experiments performed with different optical set-ups. Other applications of the quantified model include the generation of noisy images to estimate the smallest detectable signal or the 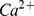
current that underlies a 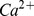
image. As far as we can tell, this is the first time that an analysis of basal fluorescence fluctuations is used for this purpose.

## Materials and Methods

### Experiments

#### Preparation of Xenopus lævis oocytes

Experiments were performed on immature *Xenopus lævis*'s oocytes previously treated with collagenase and stored in Barth's solution.

Oocytes were loaded with either Fluo-4 dextran high affinity (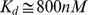) or Rhod-2 dextran (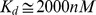), together with an exogenous 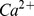
buffer, EGTA (Ethylene glycol-bis(2-aminoethylether)-N,N,N′,N′-tetraacetic acid). Intracellular microinjections were performed using a Drummond microinjector. Assuming a 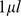 cytosolic volume, the final concentration of Fluo-4 was 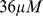
while the final concentrations of Rhod-2 were 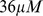
or 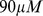
and those of EGTA 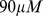
or 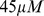. We will use lower case letters (***i***, ***ii***, ***iii***) to distinguish among the three combinations of dye type and dye and EGTA concentrations that we use in this paper as listed in Table 1.

**Table 1:** Sets of dye and EGTA concentrations.

Set	Dye type and concentration	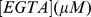
(***i***)	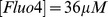	90
(***ii***)	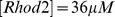	90
(***iii***)	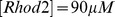	45

We also performed experiments with different (uniform) cytosolic 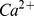
concentrations that were larger than the basal value. This was achieved by microinjecting a solution of calcium chloride (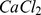). Successive microinjections of this solution were applied to a final concentration of 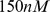
in the oocyte.

For the observation of 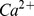
signals, caged IP_3_ was microinjected together with the dye and EGTA to a final concentration of 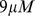.

Fluo-4, Rhod-2 and caged IP_3_ were from Molecular Probes and EGTA from Sigma Aldrich.

#### Microscopy technique

Experiments were performed using an inverted microscope IX81 connected to a multispectral confocal unit Olympus FluoView 1000 in the linescan mode. Recordings were made at room temperature. All recordings were obtained at the depth of the cortical granules in the animal hemisphere of the oocyte focusing with a 60X oil immersion objective (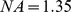). The dyes Fluo-4 and Rhod-2 were excited with the 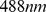 line of a multiline Argon laser and with the 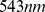 line of a He-Ne laser, respectively. The emitted fluorescence was detected in the range of 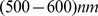 for Fluo-4 and 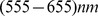 for Rhod-2 with a PMT detector. Linescan images were obtained by scanning along a fixed line (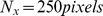) within the oocyte 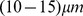. The acquisition rate was fixed at 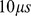
per pixel resulting in a scan rate of 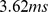
per line. Records contain a total of 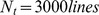. The detector was used in the photon-counting mode. In this paper we present the results of 4 types of experiments as summarized in Table 2.

**Table 2:** Type of experiments.

Experiment	Region of oocyte	Illumination power	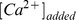	Caged IP_3_	Figures
Type 0	-	fixed (standard)	no	yes	1
Type I	variable	fixed (standard)	no	no	3A,4A,5A
Type II	fixed	variable	no	no	3B,4B,5B
Type III	variable	fixed (standard)	yes	no	3C,5C

#### Observation of IP_3_-mediated 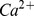
signals

For these experiments (which we call Type 0) we use oocytes previously microinjected with a 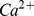
dye, EGTA and caged IP_3_. To evoke the signals, the caged IP_3_ is photolyzed with a UV pulse (of controlled duration and power) using the modification described in [21]. This modification allows the entry of ultraviolet illumination from a mercury lamp (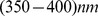) while simultaneously acquiring fluorescence images with confocal microscopy. Throughout the paper we will refer to the “standard illumination conditions” as those that are used to excite the fluorescence for the observation of IP_3_-mediated 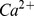
signals.

#### Experiments performed under stationary conditions

The method that we introduce in this paper is based on performing three types of experiments using the dye and EGTA concentrations of interest in oocytes where 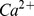
signals are not evoked (i.e., stationary conditions). We call them Type I, Type II and Type III experiments (see Table 2). This classification refers to the way the experiments are performed. We present as an example the application of our methodology to the three combinations of dye and dye and EGTA concentrations with which we try to observe 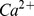 signals (sets ***i***, ***ii***, ***iii*** of Table 1). In Type I experiments, the fluorescence is collected for 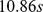 along a 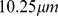 line using the same illumination power in many regions of the same oocyte. In Type II experiments, we select one region in one oocyte and obtain several 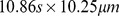 linescan images each of them for a different illumination power (the “standard” one with which 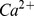
signals are observed and others). To this end, the power of the illumination beam is varied over the range that the Olympus FV1000 allows with an AOTF. In Type III experiments we microinject 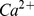
as explained before and obtain several 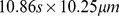 linescan images in different regions of the same oocyte and we repeat the data acquisition for different final 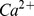
concentrations. We summarize the characteristics of the different types of experiments and list the figures obtained with each of them in Table 2.

### Image processing and analyses

**Figure 1:**
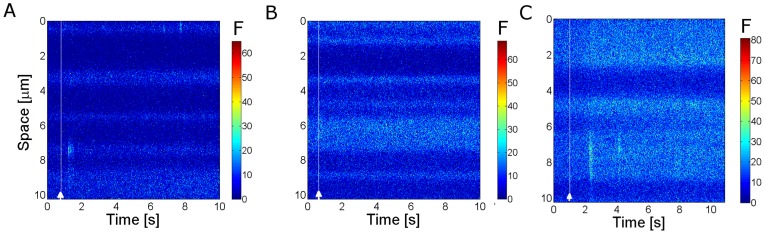
Typical row linescan images obtained in oocytes with EGTA and Fluo-4 or Rhod-2 subjected to the same uncaging conditions. (**A**) For 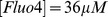, 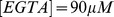, (**B**) for 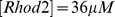
and 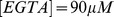, (**C**) for 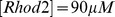
and 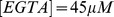. The horizontal and vertical axes correspond to time and space, respectively. The color bar represents the fluorescence intensity (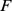). The white line marks the UV flash. In (**A**) and (**C**) several puffs are distinguishable and none can be observed in (**B**).

In this paper we present only one figure (Fig. 1) where we illustrate the fluorescence distribution obtained in Type 0 experiments. In this case, the raw fluorescence at each time, 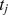, and point, 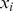, along the linescan, 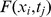, is displayed instead of the ratio defined in Eq. 1. In Fig. 1, increasing levels of fluorescence (increasing calcium levels) are represented by increasingly warmer colors.

**Figure 2:**
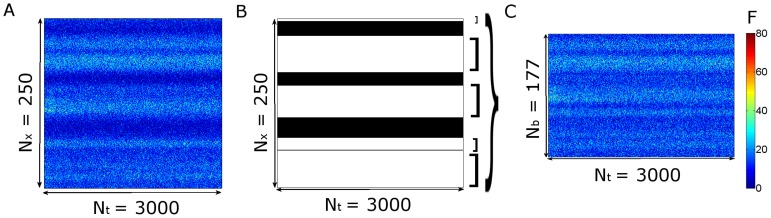
Image pre-processing applied to Type I-III experiments performed in *Xenopus lævis* oocytes. (**A**) Typical linescan image obtained with a Type I experiment where dark (cortical granules) and bright fringes (cytosol) are distinguishable. (**B**) Bright fringes (in white) and dark ones (in black) of the image in (A) identified as explained in Materials and Methods Section. (**C**) Final image once the dark fringes have been removed. Once this is done we work with all the pixels of the image without distinguishing their time or spatial coordinates. This pre-processing might not be necessary in other cell types. The color bar represents the fluorescence intensity (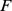) both for (**A**) and (**C**).

Most of the results of the paper correspond to Type I-III experiments. In those cases we present the results in terms of the mean and standard deviation of the fluorescence observed in the “bright” fringes (*bf*) of the analyzed records. In *Xenopus lævis* oocytes typical linescan images show horizontal lines that are persistently dark which correspond to the cortical granules (Fig. 2A). Bright fringes correspond to the cytosol. The application of our method in this case requires a pre-processing of the data so as to keep solely the information coming from the cytosol. This pre-processing, which is not necessary in other cell types, is illustrated in Fig. 2. In order to tell apart pixels corresponding to either of these two groups we first compute 
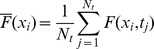
, and then we calculate the mean, 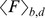, and standard deviation, 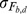, of 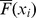 as:
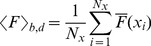
(2)

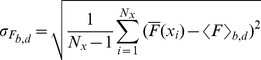
(3)


We finally identify the location of the bright spatial lines, 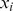, as those for which 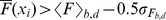
(indicated in white in Fig. 2B). Once the bright fringes are identified, we only work with the pixels of the image whose spatial coordinates correspond to those of the bright fringes (Fig. 2C).

For each linescan image obtained with Type I, Type II and Type III experiments we compute the mean, 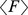, and standard deviation, 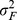, of the fluorescence of the pixels in the bright fringes:
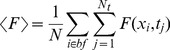
(4)

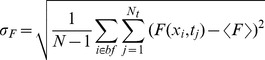
(5)where 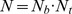 and the sum over *i* goes over the locations of the bright fringes (*bf*).

For each type of experiment (I, II and III) performed with the same dye type and dye and EGTA concentrations (sets ***i***, ***ii***, ***iii*** in the example discussed in this paper) we typically obtain between 21 and 88 linescans depending on the experiment type. We then put on the same plot the values 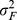 vs 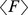 that correspond to the same type of experiment (I, II and III) applied to the same combination of dye and EGTA (e.g., ***i***, ***ii***, ***iii***). Thus, for each set (***i***, ***ii***, ***iii***) we end up having three plots of 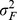 vs 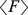. The implications and interpretation of this pooling of the data are discussed later. We fit the three plots of 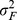 vs 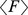 with polynomials of degree one or two depending on the type of experiment using MATLAB's cftool toolbox (The MathWorks, Natick, MA). When fitting a curve, this tool gives confidence intervals for the various fitting parameters. For the sake of simplicity, in the main body of the paper, we only quote the mean values obtained. The confidence intervals are listed in the Supporting Information (see text S1) file that accompanies the paper.

### Fluorescence fluctuations model

**Figure 3:**
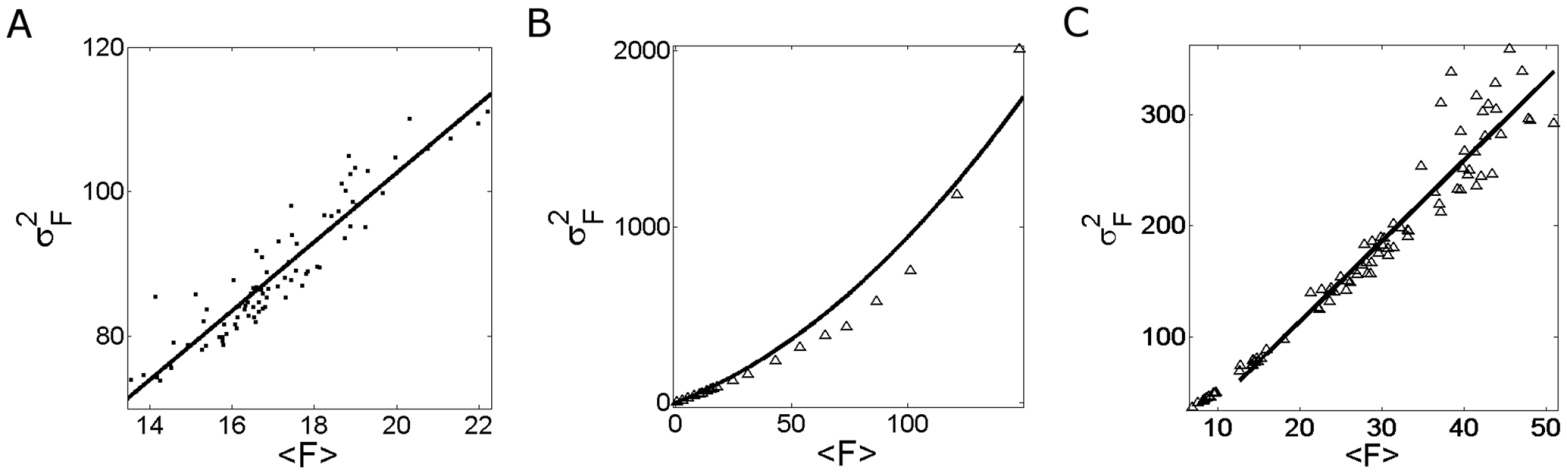
Fluorescence fluctuations obtained from Type I-III experiments performed in oocytes with the set of concentrations (*i*). The mean fluorescence (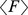) and variance (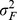) are computed as explained in Materials and Methods. The experimental data (black squares) and their corresponding fits (black line) are shown for: (**A**) 84 images obtained in Type I experiments, fit: 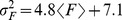; (**B**) 21 images obtained in Type II experiments, fit: 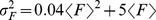; (**C**) 88 images obtained in Type III experiments, fit: 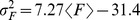.

**Figure 4:**
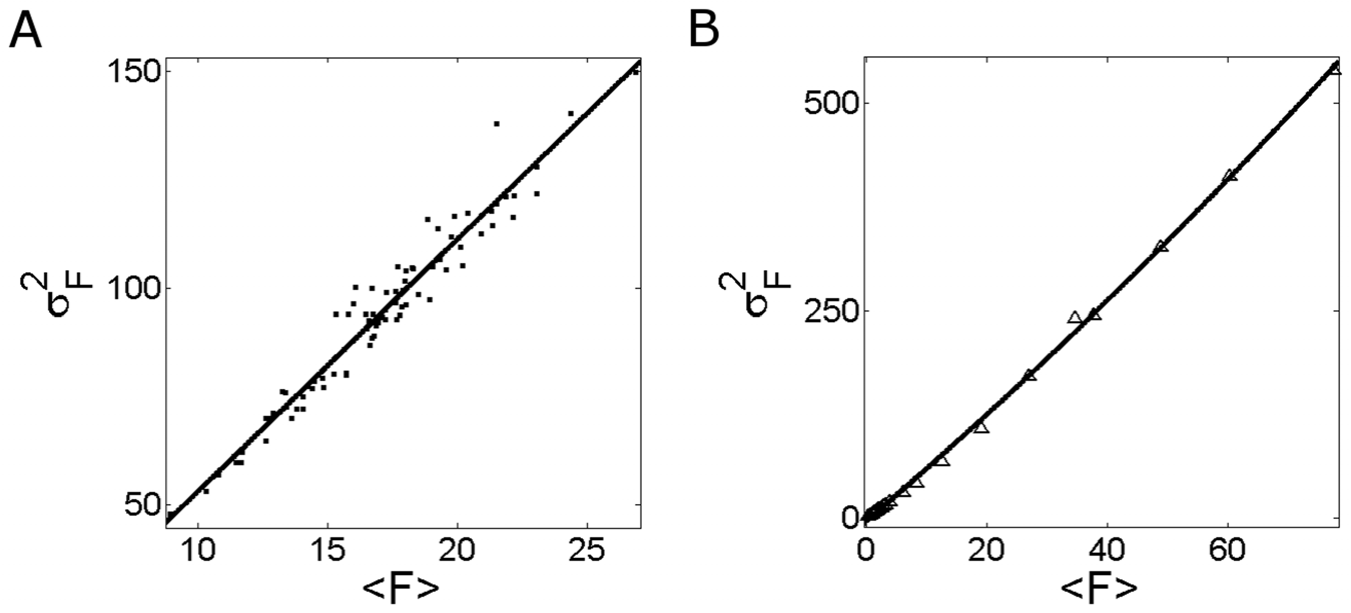
Fluorescence fluctuations obtained from Type I–II experiments performed in oocytes with the set of concentrations (*ii*). Similar to Fig. 3 but for set (***ii***). The experimental data and their corresponding fits are shown for: (**A**) 84 images obtained in Type I experiments, fit: 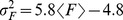; (**B**) 21 images obtained in Type II experiments, fit: 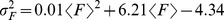. In this case the results derived from Type III experiments are not shown because no change in fluorescence was observed upon 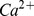
microinjection for this set of concentrations.

**Figure 5:**
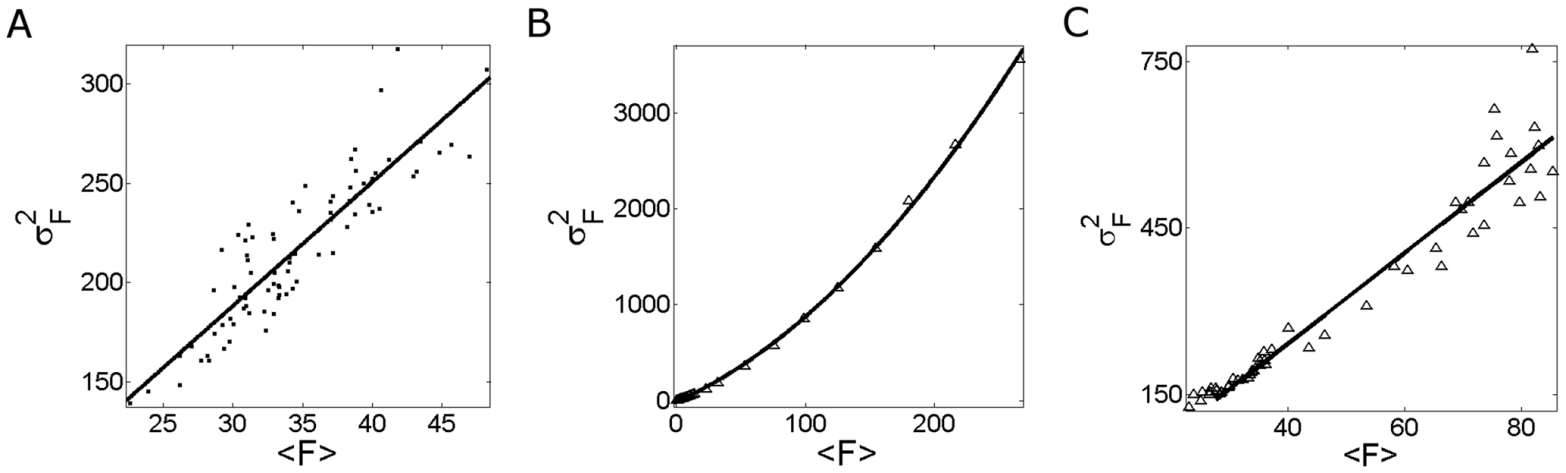
Fluorescence fluctuations obtained from Type I–II experiments performed in oocytes with the set of concentrations (*iii* *)*. Similar to Fig. 3 but for set (***iii***). The experimental data and their corresponding fits are shown for: (**A**) 84 images obtained in Type I experiments, fit: 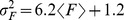; (**B**) 21 images obtained from Type II experiments, fit: 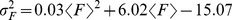; (**C**) 55 images obtained from Type III experiments, fit: 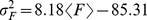.

Here we introduce the model with which we describe the fluctuations of the fluorescence collected at each pixel of a 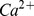 image obtained using a single-wavelength 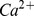
dye. With this model we analyze the fluorescence distributions obtained with Type I, Type II and Type III experiments (Figs. 3–5). By determining some key model parameters from fits to these experimental data we expect to separate three sources of fluorescence fluctuations: (1) variations in the number of dye molecules whose fluorescence is collected at each pixel, (2) changes in the fraction of such molecules that are 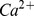-bound; (3) fluctuations in the number of detected photons. We assume that, when 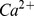
signals are evoked (Type 0), the only quantity that changes with respect to the experiments performed under stationary conditions is the probability, 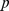, that a dye molecule be bound to 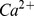 at each time and spatial point of the sample. We suggest that this probability can either be estimated roughly or computed via numerical simulations of the intracellular 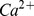 dynamics in the presence of different 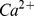
sources. Inserting in the quantitated fluctuation model the 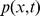 estimated for a given 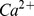
source the expected signal-to-noise ratio of the corresponding image can be computed. Repeating the approach for different combinations of dye and EGTA (in the example discussed in this paper, sets ***i***, ***ii***, ***iii*** **)** allows a direct comparison of the detectability properties of different settings in terms of their signal-to-noise ratio.

To build the fluctuation model, we extend the Number and Brightness theory [17] taking into account that, for single wavelength dyes, both the 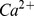-bound and the 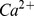-free dye molecules emit in the same range of wavelengths (albeit with a different intensity) and that the detector introduces an amplification factor. We consider that the fluorescence that is collected at each pixel of the linescan image is a random variable that depends on the pixel time and on the sources of randomness that we have already mentioned. To be more specific, let us call 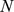 the random variable that represents the total number of dye molecules that contribute to the fluorescence at one pixel. The value that 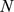 takes on at each pixel can be considered as a realization of the random variable. As done in [17] we assume that 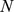 obeys Poisson statistics. Let us call 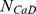 the number of dye molecules that are bound to 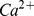 at each pixel. This is also a random variable. We will call 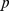 the probability that a dye molecule is bound to 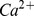. In general 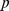 is space and time-dependent. For the experiments performed under stationary conditions we assume that 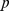
is constant and spatially uniform. This assumption is dropped for images of 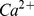
signals as explained later. In either case we assume that, given 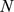, 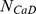 follows a binomial distribution, *i.e.* 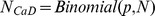. Let us assume that we can describe the emitted fluorescence with one molecular brightness for the 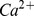-bound form of the dye and another one for its 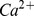-free form so that the total number of photons that reach the detector during the acquisition time to eventually give the fluorescence intensity at the pixel of interest is given by: 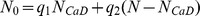. 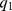
and 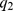 represent the number of photons per emitting molecule that reach the detector during the acquisition time for the 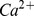-bound and 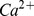-free forms of the dye. 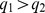 and both are increasing functions of the power of the laser.

Summarizing, we assume that the number of photons that reach the detector from the region of the sample associated to each pixel can be written as:
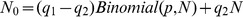
(6)with 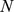
a Poisson-distributed random variable. We assume that 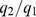 is fixed for each dye and is of the order of the ratio of the quantum efficiencies for the 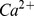 free and the 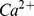-bound forms of the dye molecule. From Eq. 6 we obtain: 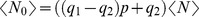 and 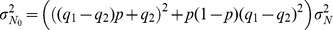 for the mean and variance of 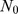, respectively. Given that 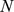 is a Poisson-distributed random variable it is 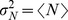. We then conclude that 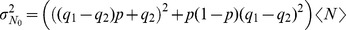. Now, the detector amplifies the signal and, at the same time, introduces some additional noise. In particular, we will assume that, if 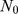 photons arrive in the detector, the fluorescence intensity that is reported at the pixel of interest is a random variable, 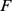, that is proportional to a Poisson distributed variable of mean 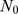, with constant of proportionality 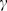, that represents the amplification factor, *i.e.*, given 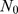, it is: 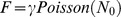. Combining this relation with Eq. 6 we obtain:
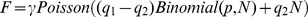
(7)with 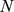 a Poisson distributed random variable.

Under these assumptions the mean and the variance (or standard deviation squared) of the fluorescence reported at the pixel are given by: 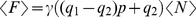
(8)

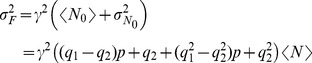
(9)


Eqs. 8 and 9 can be combined in different ways. Obtaining 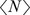 as a function of 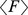, 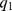 and 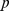
from Eq. 8 and replacing it in Eq. 9 we obtain:
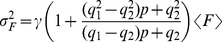
(10)


Using Eq. 8 to write 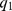 as a function of 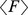, 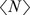 and 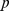
and inserting it in Eq. 9 we obtain:
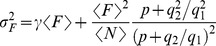
(11)


Finally, using Eq. 8 to write 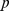
as a function of 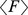, 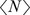 and 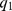 and inserting it in Eq. 9 we obtain:
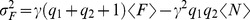
(12)


We will use either one of these last equations to interpret and fit the 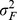 vs 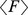 plots obtained with Type I–III experiments. The expression that is used in each case depends on which quantities can be assumed to have the same values for the data points that are pooled together in each type of experiment (see Table 3). As explained in more detail in the Results Section, the combination of the fits to the three types of experiments allows the quantification of 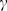, 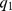 at the standard illumination power and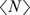. Once we have 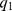, we use the ratio of quantum efficiencies of the 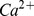 free and the 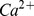-bound dye molecules to estimate 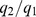
and determine 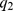. We expect 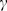 to depend only on the way in which the fluorescence is detected to generate the image, 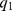 and 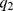 to depend on the quantum efficiency of the dye and on the laser power and 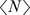 to depend on the dye concentration. Actually, we allow for 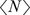 to vary among different regions of the same oocyte. Therefore, with the method we estimate a range of possible 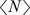 values. Now, 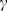, 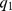 and 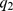 at the standard illumination power and 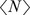 should remain approximately the same for any experiment (including those of Type 0) that is performed using the same experimental set-up, the same dye, in the same concentration and with the same illuminating laser power as those performed under stationary conditions (Type I–III) to estimate them. Thus, we only need the fraction of 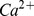-bound dye molecules at each spatial point and time during a 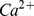
signal to obtain the expected signal-to-noise ratio for each experimental setting. As explained in what follows this fraction can be obtained by means of numerical simulations of the intracellular 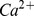
dynamics.

**Table 3:** Behavior of the fluctuation model parameters in the experiments performed under stationary conditions.

Experiment	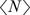	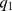	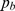
Type I	variable	fixed	-
Type II	fixed	variable	fixed
Type III	variable	fixed	variable

### Numerical simulations

#### Determination of the 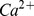-bound dye distribution in the presence of a localized 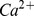 source

Numerical simulations of the calcium dynamics in the cytosolic medium are performed solving a set of reaction-diffusion equations in a spherical volume, assuming spherical symmetry, for the following species: 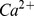, an immobile endogenous buffer (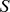), a cytosolic 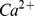 indicator (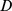) and an exogenous mobile buffer (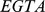). In some cases we also consider an additional (mobile) buffer (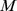). A point source of calcium located at the origin and pumps (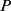) that remove calcium uniformly in space are also included. The source represents a cluster of IP_3_Rs.

We consider that a single 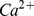
ion binds to a single buffer or dye molecule according to:
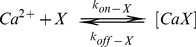
(13)where 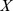 represents 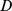, 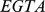, 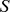 or 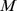, and 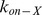
and 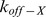
are the forward and backward binding rate constants of the corresponding reaction, respectively. We assume that the total concentrations of dye, EGTA, mobile and immobile buffer remain constant (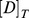,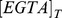,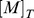, and 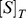, respectively) and that the diffusion coefficient of their free and 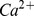 bound forms are equal. Therefore we calculate the free concentrations, 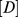, 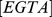, 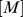 and 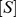 by subtracting the concentration of their 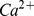 bound forms to their total concentrations. Given these assumptions, the set of reaction-diffusion equations reads:



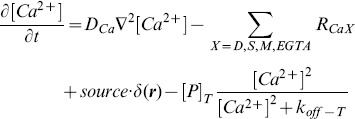
(14.a)

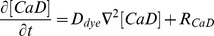
(14.b)

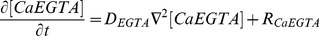
(14.c)

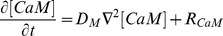
(14.d)

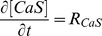
(14.e)where 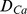, 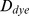, 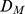
and 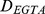 are the diffusion coefficients of 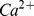,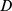, 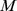
and 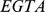, respectively. The reaction terms, 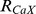,are derived from the kinetic scheme, Eq. 13.



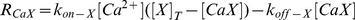
(15)We assume no flux boundary conditions at 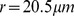 with 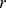 the radial coordinate. For the source we assume that it consists of 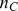 channels that open simultaneously at 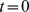 each of which closes after a time that is drawn from an exponential distribution with mean 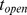 22]. For the initial condition, we assume that all concentrations are homogeneously distributed, 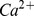 is at basal concentration and all species are in equilibrium among themselves (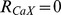
for all 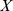 (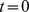)).

The reaction-diffusion equations are solved using a backward Euler method in time and an explicit finite-difference formula in space with a 2^nd^ order expression (first neighbors) for the Laplacian. The spatial grid size is 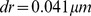 and the time step 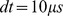. The values of the parameters (taken from [18]) used in the simulations are listed in Table 4. To compare with experimental confocal signals we calculate a weighted average of 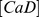 along the linescan 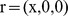 according to the confocal microscope point spread function (PSF). In this case, following [23], the confocal signal as a function of 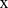 is:
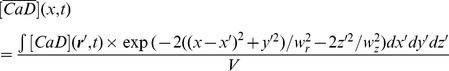
(16)where 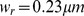, 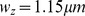 and 
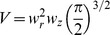
. This is the blurred version of the 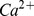-bound dye concentration. Having 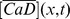 we can compute the space and time dependent probability that a dye molecule is bound to 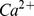 during a 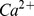
signal as 
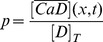


**Table 4:** Parameter values used to solve the simulations introduced in Materials and Methods Section.

Parameter	Value	Units
Free calcium:		
*D_Ca_*	220	µm^2^s^−1^
[*Ca^2+^*]*_basal_*	0.1	µM
Calcium dye Fluo-4 dextran:		
*D_dye_*	15	µm^2^s^−1^
*k_on-D_*	240	µM^−1^s^−1^
*k_off-D_*	180	s^−1^
[*D*]*_T_*	36	µM
Calcium dye Rhod-2 dextran:		
*D_dye_*	15	µm^2^s^−1^
*k_on-D_*	70, 85	µM^−1^s^−1^
*k_off-D_*	130, 170	s^−1^
[*D*]*_T_*	36, 90	µM
Exogenous buffer EGTA:		
*D_EGTA_*	80	µm^2^s^−1^
*k_on-EGTA_*	5	µM^−1^s^−1^
*k_off-EGTA_*	0.75	s^−1^
*[EGTA]_T_*	45, 90	µM
Endogenous immobile buffer:		
*K_on-I_*	400	µM^−1^s^−1^
*K_off-I_*	800	s^−1^
[*I*]*_T_*	300	µM
Slow endogenous mobile buffer:		
*D_M_*	27	µm^2^s^−1^
*K_on-M_*	20	µM^−1^s^−1^
*K_off-M_*	8.6	s^−1^
[*M*]*_T_*	250	µM
Rapid endogenous mobile buffer:		
*D_M_*	32	µm^2^s^−1^
*K_on-M_*	500	µM^−1^s^−1^
*K_off-M_*	750	s^−1^
[*M*]*_T_*	10	µM
Pump:		
*K_off-P_*	0.1	s^−1^
[*P*]*_T_*	0.9	µM
Source:		
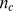	6	-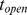	20	ms

### Simulated fluorescence distribution

We generate noisy simulated images using the model described in the previous Subsection. Namely, for each position, 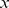, and time, 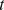, of the simulation, we draw a stochastic variable, 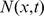, from a Poisson distribution whose mean is given by the value, 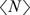, estimated from the experiments. We then compute the fluorescence at each pixel, 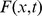, following Eq. 7, with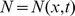, 
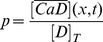
 (with 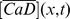 obtained from the simulations as defined in Eq. 16) and the values of 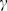, 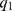 and 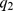 estimated from the experiments.

For each noisy simulated image we compute the signal-to-noise ratio as:
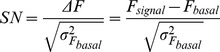
(17)with 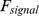 and 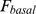 the maximum and minimum fluorescence values of the simulated image, respectively, and 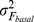
computed using Eq. 9 with 
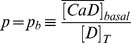
. An estimate of the signal-to-noise ratio can also be obtained replacing 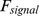 and 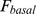 in Eq. 17 by the mean values given by Eq. 8 with 
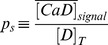
 and 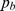, respectively. The value of 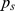 can in turn be computed using the maximum expected value of the 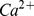-bound dye concentration during the signal. In this way, the signal-to-noise ratio can be written as:
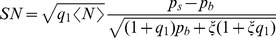
(18)neglecting terms of the order of 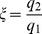 in front of 1. Eq. 18 can be re-written in terms of quantities that are straightforwardly related to experimentally accessible parameters. In particular, assuming that 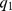 is directly proportional to the intensity of the laser, 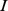, we can rewrite 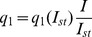, with 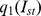 the value of 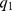 at 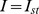, the intensity of the laser at the standard illumination power. Replacing 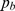 as a function of 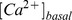 we can write it as 
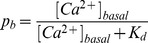
. In this way, we can compute the SN ratio as a function of 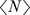, 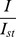 and 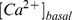:
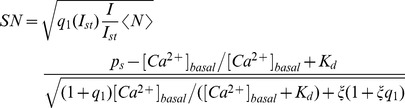
(19)


## Results

In the Materials and Methods Section we introduced a method to derive a quantitative description of the fluorescence fluctuations of images obtained using single-wavelength 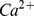
dyes. In this Section we derive this quantitative description for three situations (sets (***i***)–(***iii***) of Table 1). The first one corresponds to a choice of dye and concentrations that has proven to be adequate for the observation of 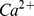 puffs [21], (set (***i***)). The other two involve using a dye that has been less characterized for the observation of this type of signals, Rhod-2 (sets (***ii***) and (***iii***)). We have chosen these three sets to show that the fluctuation model can be used to classify 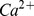
imaging experimental settings and that the classification is able to discriminate between experimental settings with clearly different signal-to-noise ratios (when probed with Type 0 experiments). The differences in the signal-to-noise ratios for these three situations are apparent in Fig. 1. We show in this figure linescan images obtained in oocytes after photoreleasing IP_3_ with the same uncaging pulse (the occurrence of which is marked with a white line in the images). Fig. 1A corresponds to set (***i***), Fig. 1B to set (***ii***) and Fig. 1C to set (***iii***). Puffs can be observed in Figs. 1A and 1C, but not in Fig. 1B. In fact, we repeated the experiments for the conditions of Fig. 1B in many different oocytes and could never observe isolated signals using Rhod-2 and EGTA at these concentrations. As we show in this Section, our method puts the experiments of Figs. 1A and 1B in two separate classes. It also predicts that the signal-to-noise ratio of the experiment in Fig. 1C should be similar to that of Fig.1A. More interestingly, the numerical simulations show that this separation in two different classes is not achieved if one compares the 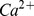-bound distribution without including the various sources of fluctuations that our method discriminates. In this Section we also discuss the rationale for analyzing the data of the various experiments in the way that we do it.

### Outline of the method to quantitate the contribution of the different sources of fluorescence fluctuations in images obtained using single-wavelength 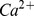
dyes

Before advancing with the particular application of our method to sets (***i***)–(***iii***), we first outline how to proceed in a generic situation. Let us suppose we want to obtain images of 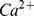 signals using a single-wavelength dye (e.g., we want to perform an experiment of Type 0) and that we wish to evaluate *a priori* the performance of the experimental set-up for a certain combination of dye, illumination power and dye and EGTA concentrations (i.e., for an *experimental setting*). Then, we microinject the desired quantities of dye and EGTA in different oocytes and perform Type I, Type II and Type III experiments as described in the Materials and Methods Section. For each experimental type we process the data as described before and obtain plots of fluorescence variance, 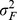, as a function of mean fluorescence, 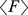. For classification purposes a simple possibility is to compare the three curves obtained for the dye of interest with those obtained for another dye or at other concentrations at which it is known that 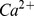 puffs can be observed. Experiments performed with different set-ups can also be compared. For a more quantitative comparison we use one among Eqs. 10–12 to fit each of the three 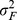
vs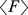 curves obtained experimentally. We discuss in the next Subsection what equations are applicable in each case. The three fits should serve to quantify the parameters 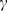, 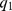 and 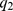 (at the standard illumination power) and to determine a range of values for the mean number of dye molecules, 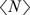 for the experimental settings that are being probed. When using the same experimental set-up and the same combination of dye type, dye and EGTA concentrations, these four parameters should remain approximately the same for Type 0 and for Type I-III experiments. The signal-to-noise ratio during a 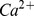
signal (i.e., in a Type 0 experiment) depends on these four parameters and on 
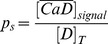
 (see Eq. 18). Thus, once the four parameters are determined with Type I-III experiments, it is only necessary to have an estimate of the fraction of dye molecules that are bound to 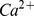
at the location of the signal maximum in order to compute the signal-to-noise ratio that can be expected in a Type 0 experiment. This fraction can be estimated roughly or by means of numerical simulations, as explained in Materials and Methods Section. This outline shows that the application of our method allows an *a priori* (quantitative) estimate of the signal-to-noise-ratio for a given experimental setting. We now describe in detail the application of this approach to the sets (***i***)–(***iii***) explored in Fig. 1.

### Fluctuation analysis of experiments with 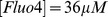
and 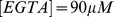


We show in Figs. 3 A, B and C the variance of the fluorescence, 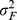, as a function of its mean, 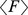, derived from Type I-III experiments performed on oocytes microinjected with the set of concentrations (***i***) of Table 1.

Fig. 3A collects the data obtained in several regions of the same oocyte with the standard illumination power and without 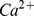
microinjection (Type I see Tables 2 and 3). Thus, 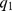 and 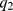 take on the same values for all the data points of Fig. 3A. The fraction of 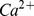-bound dye molecules, 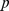, could vary from region to region due to a non-uniform distribution of basal cytosolic 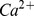. We observe in Fig. 3A, however, that 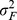 and 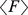 are linearly related and that the 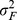 vs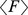 curve practically goes through the origin (the ordinate to the origin given by the fit is an order of magnitude smaller than the values of 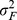 obtained in the experiments). Given Eq. 10, this is expected if variations in the basal value of 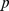 (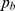) in different regions of the same oocyte do not have a noticeable effect on the ratio 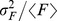. We then use Eq. 10 to describe the curve displayed in Fig. 3A and equate 
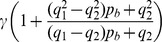
 to the slope of the linear fit, *i.e.* 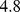. This is the first piece of quantitative information that we derive from the experiments. It is implicit in this interpretation of the data that different points along the 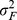 vs 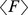
curve have different values of 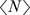 which is consistent with having slightly different dye concentrations in different cytosolic regions of the same oocyte (Table 3).

We show in Fig. 3B the plot of 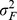 vs 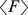 derived from the analysis of linescan images obtained in Type II experiments. In this case, since we do not pool together data coming from different regions, we assume that 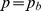 and 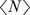 take on approximately the same value for all data points (see Table 3). Regarding 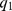
and 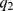 we can only assume that their ratio is constant but not their individual values since we are varying the laser power. We then use Eq. 11 to interpret the fits to the data. In particular, fitting the data points with a second degree polynomial we derive 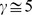. Comparing these estimates with the slope derived from the fit of Fig. 3A we conclude that 
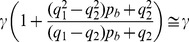
 for the experiments of Fig. 3A. Thus Eq. 10 reduces to:
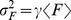
(20)under basal conditions for the standard illumination power used in Fig. 3A. The estimates of the amplification factor 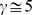 also agree with an analysis of the detectors behavior in the photon counting mode and in the absence of a fluorescence stimulus (data not shown). In particular, after subtracting the zero counts, the mean number of counts reported by the detectors in darkness is consistent with this amplification factor, namely, it is 
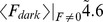
. If the zero counts are included as well we find 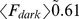 which shows that the detectors do not introduce a noticeable offset in the reported fluorescence.

We show in Fig. 3C the plot of 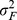
vs 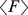 derived from the analysis of linescan images obtained with experiments performed using the same (standard) illumination power as in Fig. 3A but in oocytes microinjected with dye, EGTA, and different amounts of 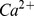 to change the cytosolic 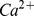 concentration, 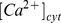. Data coming from the same batch of oocytes but with no added 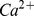
are also included in the plot. In Fig. 3C not all the data points correspond to the same value of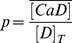. Furthermore, the value of 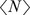 cannot be assumed to be the same for all the points either (see Table 3). In any case, if 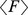 is large enough so that 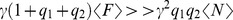, Eq. 12 reduces to: 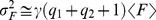
(21)


A similar relationship is obtained from Eq. 10 if 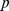 is large enough so that 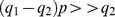, or equivalently, 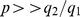 (for situations like those analyzed in this paper in which 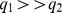). Thus, using Eq. 21 to fit the points with large enough 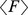 of Fig. 3C we estimate 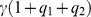. We obtain 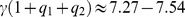 depending on how many points we keep to do the fit (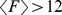 as illustrated in Fig. 3C or 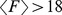, respectively). Using the values of 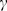 estimated from Fig. 3B and assuming that 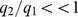 we derive 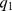 from these fits. We obtain 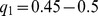. Assuming that 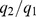 is of the order of the ratio of quantum efficiencies estimated for Fluo-3 in [24], 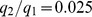, we can derive 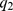 as well. We obtain 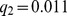 for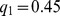. These values correspond to the standard illumination power since they are derived from Type III experiments (see Table 2).

From Fig. 3A a range of possible mean values, 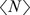, can be estimated using Eq. 8 with the previously inferred values, 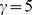, 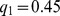 and 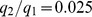. In particular, using 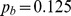 (which corresponds to 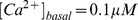 and 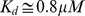) we find that the range of mean fluorescence values of Fig. 3A (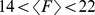) corresponds to 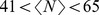. If we consider 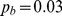, instead, (which corresponds to 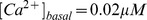) we obtain 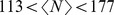.

Summarizing, from the fits analyzed so far we have estimated 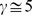, 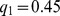, 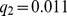 and a range of 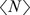 for different values of 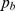 for set (***i***).

### Rough estimate of the expected signal-to-noise ratio for the observation of 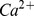 puffs for experiments with 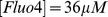
and 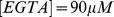


Having obtained 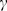, 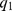, 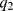 and 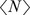 we are in a position of estimating the signal-to-noise ratio as a function of the fraction of 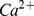-bound dye, 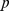, during a signal. Here we only give a rough estimate. A more accurate description can be obtained with numerical simulations as described later. To this end we use Eq. 18. Given the previous estimates of the model parameters we obtain 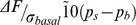 for 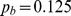 and 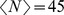. Thus, already at 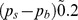 it is 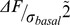 and we expect the signal to start to be distinguishable. An estimate of the underlying 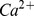
source that produces a difference 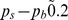 can be obtained with numerical simulations.

### Fluctuation analysis of experiments performed with Rhod-2

We now repeat the experiments and analyses described in the previous Subsections but for oocytes microinjected with two sets of Rhod-2 and EGTA concentrations: set (***ii***) with the same dye and EGTA concentrations as in set (***i***) and with set (***iii***) with a larger dye and smaller EGTA concentrations. Given that the same laser and the same standard illumination conditions are used for sets (***ii***) and (***iii***), we expect that the fluctuation model should be characterized by the same values of 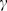, 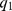 and 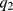 at the standard illumination power. The value of 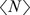, however, should be different due to the different values of 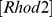.

We show plots of the variance 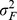, as a function of the mean fluorescence, 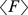, derived from Type I–II experiments performed using set (***ii***) in Figs. 4A and 4B and from Type I–III experiments performed using set (***iii***) in Figs. 5A, 5B and 5C.

We show in Figs. 4A and 5A the plots derived from Type I experiments. We observe that for both sets of concentrations 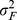 and 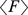 are linearly related and that the 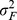
vs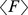 curve goes through the origin. Furthermore, for both types of experiments the fit to the data approximately gives the same slope 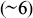. Thus, 
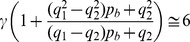
 for both types of experiments which is a consequence, as in the case of Fluo-4, of the fact that the second term inside the parenthesis is much smaller than one.

We show in Figs. 4B and 5B data points obtained from Type II experiments. As done for Fluo-4 we assume that 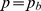 and 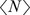 take on approximately the same value for all the points of each figure and that the ratio, 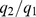, but not the individual values, 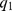 and 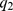, is constant. In this case we observe a fundamental difference between the plots of Figs 4B and 5B. Namely, while the 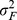
vs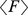 curve has a parabolic shape in Fig. 5B, it remains a straight line in Fig. 4B. This latter behavior is different to the one observed for Fluo-4 too (Fig. 3B). Using either a linear or second degree polynomial to fit the points of Fig. 4B and interpreting the results with Eq. 11 we derive 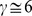. We obtain a similar value from the fit (in this case, a second degree polynomial) of the points in Fig. 5B. We then conclude that for the experiments performed with Rhod-2 (that use the He-Ne laser) it is 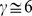. The different behavior observed in Figs 4B and 5B can then be attributed to the fact that the nonlinear part of the 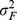 vs 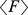 curve is not reached for the laser powers probed in case (***ii***). We discuss later possible causes for this different behavior which can be used to discard combinations of dye and EGTA concentrations that are not good for the visualization of localized 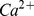
signals.

We show in Fig. 5C the data points obtained from experiments of Type III. We do not show the corresponding figure for case (***ii***) because we did not observe any variation of the fluorescence upon 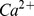 injection. The reason that underlies the different behaviors observed in Figs. 4B and 5B may also underlie the lack of fluorescence variations observed upon 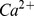
microinjection for case (***ii***). From the slope of a linear fit to the points with large enough 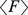 of Fig. 5C we estimate 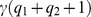 as before. Using 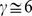 and assuming that 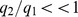 we obtain 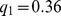 at the standard illumination power.

We now estimate the range of possible mean values, 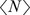, from the data of Figs. 4A and 5A. To this end, we use Eq. 8 with 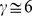, 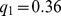 and 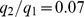. In particular, for case (***ii***) from Fig. 4A, we obtain 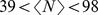 using 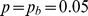 (which corresponds to 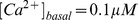) and 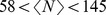 using 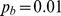 (which corresponds to 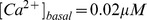).These values are consistent with the ones derived for Fluo-4 at the same concentration of dye and EGTA (Fig. 3A). For case (***iii***) we obtain, from Fig. 5A, 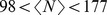 for 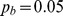
and 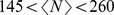 for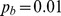. These values of 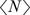 are between 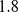 and 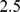 times larger in Fig. 5A than in Fig. 4A.

**Figure 6:**
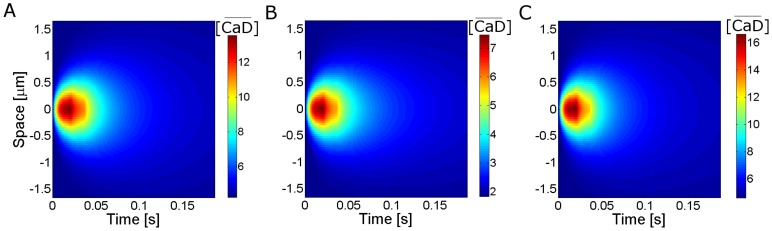
Simulated 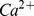-bound dye concentration during a Type 0 experiment in a linescan image. sBlurred 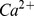-bound dye concentration obtained for: (**A**)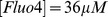, 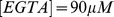
(set (***i***)); (**B**) 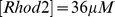, 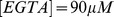
(set (***ii***)); (**C**) 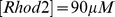, 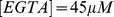
(set (***iii***)). All other parameters are as given in Table 4. In all the simulations a puff involving the simultaneous opening of 6 IP_3_Rs, 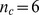, occurs at time 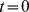.

We now estimate the signal-to-noise ratio as a function of the fraction of 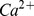-bound dye, 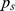, during a signal (i.e., the expected value during a Type 0 experiment). We again use Eq. 18. Setting 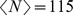 and 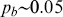 for set (***iii***) we obtain 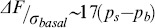. Given the different kinetic properties and dissociation constant between Fluo-4 and Rhod-2 we do not know *a priori* by how much the 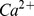-bound dye concentration in the presence of the same source would differ for set (***i***) and for sets (***ii***) or (***iii***). This can be estimated by means of numerical simulations. In particular, numerical simulations of the intracellular 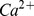 dynamics when there is a localized 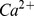
source performed as explained in Materials and Methods Section show that the ratio 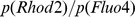 is slightly larger at the peak of the signal than at basal conditions when simulations for sets (***i***) and (***iii***) are compared (see next Subsection). In particular, for the simulations displayed in Figs. 6A and 6C it is 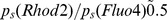 while 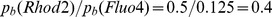. Using these estimates we conclude that the source that would give a signal-to-noise ratio of the order of 2 for Type 0 experiments performed with set (***i***) 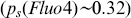 corresponds to 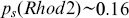 (for Type 0, set (***iii***)) experiments. Inserting 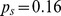 and 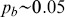 into 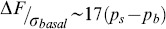 we determine that such a source would give a signal-to-noise ratio of the order of 1.9 for Type 0 experiments performed with set (***iii***).

This estimate drops by a factor of 1.6 if 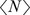 is decreased to the values that can be expected for the set (***ii***). Thus, based on the rough estimates of the signal-to-noise ratios derived so far, the application of our method puts the experiments of case (***i***) in the same equivalence class as those of case (***iii***). So, in principle, localized 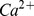
signals should be equally detectable with either one of these experimental settings. The smallest concentration of EGTA used in the latter could be compensating the larger dissociation constant of Rhod-2 and be the cause for this to happen. In fact, we prove that this is the case with numerical simulations that allow us to estimate 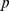 for a very small signal. According to these rough estimates the set of concentrations (***ii***) belongs to a different class.

### Numerical simulations of the model to compute signal-to-noise ratios of Type 0 experiments and establish equivalence classes

**Figure 7:**
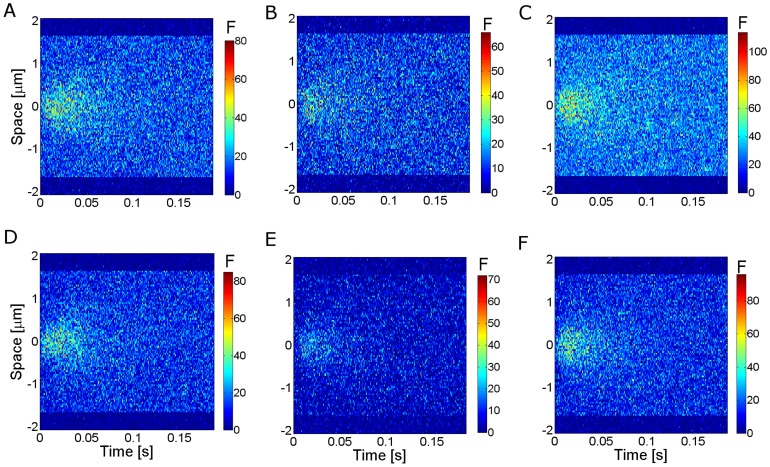
Simulated fluorescence during a Type 0 experiment in a linescan image. The fluorescence is computed from the 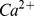-bound dye distributions of Fig. 6 as explained in Materials and Methods Section. (**A**) and (**D**) correspond to set *(* ***i*** *)*, (**B**) and (**E**) to set (***ii***) and (**C**) and (**F**) to set (***iiii***). (**A**), (**B**) and (**C**) are obtained using 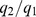 equal to the ratio of quantum efficiencies estimated in [22]. (**D**), (**E**) and (**F**) are obtained setting 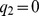, *i.e.*, they do not take the 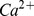
–free dye fluorescence into account. In all cases the values of 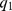 are the ones derived from the fluctuation analyses for the standard illumination power.

We now show the results of using numerical simulations to compute the expected signal-to-noise ratio for the experimental conditions explored in Fig. 1. For this particular example we simulated the 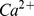, dye and buffer dynamics as described in Materials and Methods Section using the parameters of Table 4 but without including the mobile endogenous buffers listed in that Table. We used the same 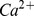
source in the three cases. In particular, we chose a situation with 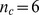 IP_3_Rs that were initially open and subsequently closed randomly with mean time, 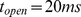. We have chosen this source because it gives signal amplitudes of the order of the smallest detectable one (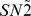). We explored other sources obtaining similar results as those illustrated in this Section (data not shown). We show in Fig. 6 the blurred 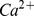-bound dye distributions obtained with the simulations where (A) corresponds to set (***i***), (B) to set (***ii***) and (C) to set (***iii***). Once we had these noiseless plots, we replaced the value of the 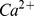-bound dye concentration at each “pixel” by a random fluorescence value as explained in in Materials and Methods Section. We show the resulting noisy images in Fig. 7. To obtain more realistic images we also added dark fringes that correspond to the granules in the oocyte. Figs. 7A and 7D correspond to simulations with set **(** ***i*** **)**, Figs. 7B and 7E to set (***ii***) and Figs. 7C and 7F to set (***iii***). In order to go from the 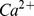-bound dye to the fluorescence distribution we used 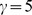 and 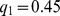 for Fluo-4 and 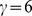 and 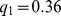 for Rhod-2, as derived from the previous analyses. For set (***iii***) (which has 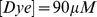), we chose 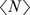 among the values estimated from Fig. 4C, namely, 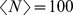. For comparison purposes, we chose 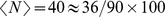 for sets (***i***) and (***ii***) (which have 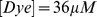). Figs. 7A-C were obtained using values of 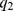 such that their ratio with respect to 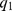
was equal to the ratio of quantum efficiencies estimated in [24], *i.e*., 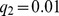 (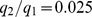) for Fluo-4 (set (***i***)) and 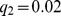 (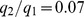) for Rhod-2 (sets (***ii***) and (***iii***)). Figs. 7D–F were done using 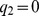 to analyze the role of the 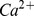-free dye fluorescence on the signal-to-noise ratio for this particular example (see Discussion). Comparing Figs. 7A–C we observe that the puff is less distinguishable when using Rhod-2 and EGTA at the same concentrations as in the simulations with Fluo-4 (Figs. 7A and 7B, respectively). The puff becomes distinguishable when the concentration of Rhod-2 is increased and that of EGTA is decreased (Fig. 7C). These results agree with the experiments of Fig. 1. These qualitative observations are quantified in the second column of Table 5 where we show the signal-to-noise ratio computed as explained in Materials and Methods Section. There we see that the signal-to-noise ratio is smallest for the set of concentrations (***ii***) and that the one obtained for the set (***iii***) is only slightly smaller than the one obtained for the set (***i***) so that a similar level of detectability of 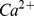
signals can be expected for these two experimental conditions (in Type 0 experiments). Thus, the numerical simulations of the model confirmed our previous rough comparison between the experimental settings explored in Fig. 1 putting in the same equivalence class the conditions of Fig. 1A and Fig. 1C and in a different one those of Fig. 1B.

**Table 5:** 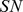
 ratios obtained in the noisy simulations.

Set	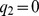	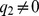
(***i***)	2.7	2.5
(***ii***)	2.3	1.5
(***iii***)	3.1	2.1

### Using the model to choose adequate experimental parameters

**Figure 8:**
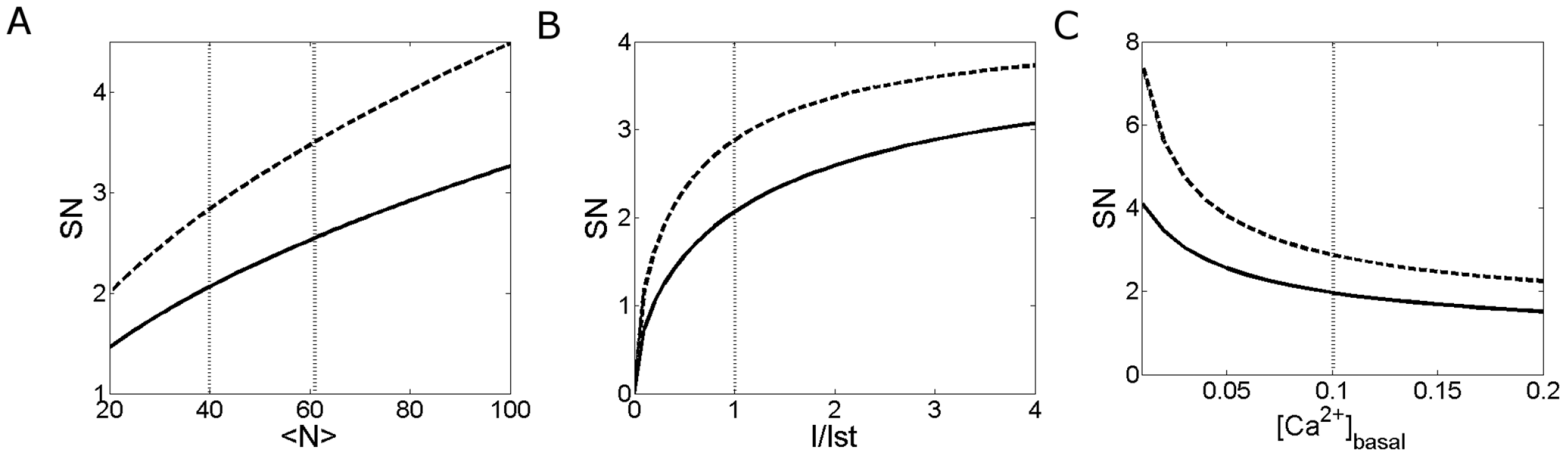
Dependence of the signal-to-noise ratio on experimentally accessible parameters. Plots of the signal-to-noise ratio using Eq. 19 with the parameter values determined for Fluo-4 (solid line) and for Fluo-8 (dashed line) as a function of the mean number of dye molecules, 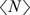 (**A**), the normalized laser intensity, 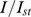 (**B**) and 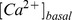 (**C**). The vertical dotted lines indicate the range of 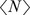 values and the values of 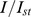 and 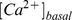 that correspond to set (***i***) at the standard illumination power. See text for more details.

Once the model is quantified for a certain dye, standard illumination power and dye and EGTA concentrations, it can then be used to determine how changes in these experimental parameters affect the expected signal-to-noise ratio. As expected, it follows from Eq. 18 that the signal-to-noise ratio increases with 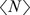 (i.e., with the dye concentration) and with 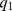 (i.e., with the illumination power or, equivalently, the laser intensity, 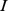) while it decreases with 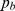 (i.e., the concentration of basal 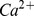, 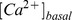). The quantified fluctuation model, however, gives more information than that. Namely, it can be used to select the experimental conditions in a more quantitative way. As an example, we show in Fig. 8 plots of the signal-to-noise ratio, given by Eq. 19, as a function of 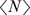 (A), 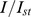 (B) and 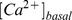 (C) for two dyes. The aim of this figure is two-fold. First, we illustrate how to use our method to select good experimental conditions for a given dye (in the example, Fluo-4). The second goal is to illustrate how to select good experimental conditions when planning the replacement of a dye by another that is excited with the same laser but that has different photo-physical properties (in the example, Fluo-4 and Fluo-8, respectively). This approach can be applied to ensure comparability between experiments performed with the “old” and the “new” dye. For these plots we first computed the signal-to-noise ratio using Eq. 19 with the values 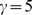, 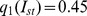 and 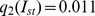, determined from the application of our method to set (***i***) and 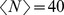 in (B) and (C), the standard illumination intensity, 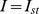, in (A) and (C) and 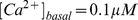 in (A) and (B). For these three curves (shown with solid lines in the figure) we computed the signal-to-noise ratio assuming that the fraction of dye molecules that are bound to 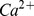 at the location of the signal maximum is 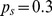 (the smallest detectable signal amplitude according to the previous discussion in Result Section). The vertical dotted lines indicate the range of 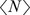 values determined from the application of our method to set (***i***) in Fig. 8A, the standard illumination condition, 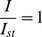, in Fig. 8B, and, in Fig. 8C, the value of 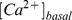 we have been using for set (***i***), 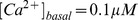 (which corresponds to 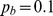, 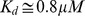). From the observation of the solid line curves in Fig. 8 we conclude that moderate increments in 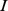 would not result in noticeable changes in the signal-to-noise ratio of Type 0 experiments performed with Fluo-4. Something similar happens with 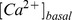, a parameter whose change would directly affect the type of signals that can be evoked. The natural variations in 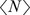 that can be encountered by changing the region of the oocyte where the experiments are performed, on the other hand, can lead to a ∼20% improvement in the signal-to-noise-ratio.

The dashed curves in Fig. 8 are the equivalent of the solid line ones but for Fluo-8. In order to compute them we used 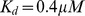 and assumed that Fluo-8, when bound to 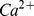 and excited with a 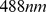 wavelength, is approximately twice as bright as Fluo-4 while the brightness of the 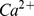-free form of both dyes is approximately the same (see *e.g.* Fluo-8 data specification sheet, Teflabs). The last two assumptions imply that we replaced 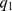 by 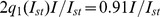 and 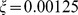 when going from Eq. 18 to Eq. 19. We estimated the value of 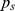 for Fluo-8 simply by assuming that it would differ from the value used for Fluo-4 (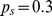) as if 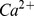 and the dye were at equilibrium at the peak of the signal. Namely, we determined 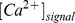 at the peak from 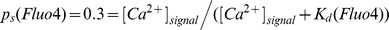 and then computed 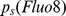 using the same formula but with 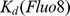. We obtained 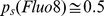. As in the case of Fluo-4 we used 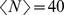 in (B) and (C), the standard illumination intensity, 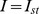, in (A) and (C) and 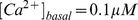 in (A) and (B). From a comparison of the solid and dashed curves we can then estimate by how much experimentally accessible parameter values should be changed in order to obtain equivalent signal-to-noise ratios with the old (Fluo-4) and the new dye (Fluo-8). It can also be used to determine the expected difference in signal-to-noise ratios when using both dyes in the same concentration.

## Discussion and Conclusions

Optical techniques and 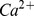 fluorescent dyes offer the possibility of observing intracellular 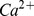
signals with minimum disruption. The fluorescence changes that occur during signals are related to the 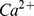
–bound dye concentration, a quantity that not only depends on free 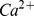 but also on the ability of the dye to overcome other 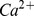 trapping mechanisms such as buffering. Furthermore, when trying to observe IP_3_-evoked localized 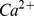
signals, exogenous buffers such as EGTA are typically introduced in the cell to disrupt the 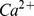-mediated communication between clusters of channels. This additional buffer also competes with the dye for 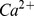
and can degrade the signal. There is great interest in studying local IP_3_R-mediated 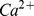
signals (puffs) since they constitute the building blocks of more global signals. Puffs are highly stochastic. In order to have an accurate statistical description of their properties it is necessary to have experiments that report the occurrence of puffs with similar levels of accuracy over the whole range of event sizes. Being able to estimate the signal-to-noise ratio that can be expected from an experimental setting can at least provide information on the reliability of the event size distribution in the region of small events. In this paper we have introduced a method that provides an *a priori* estimate of the signal-to-noise ratio of experiments that use single wavelength 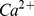
dyes and EGTA. These are the dyes that are used to observe IP_3_R-mediated 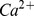
signals that are evoked via the photorelease of caged IP_3_ with UV illumination.

Our approach to probe the performance of 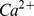-imaging experiments is based on the Number and Brightness (N&B) method of [17], [25]. It entails performing a series of experiments under stationary conditions (Type I–III) from which the parameters of a model of fluorescence fluctuations can be quantified. Combining the quantified fluctuation model with simulations of the dynamics of intracellular 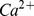
in the presence of a localized 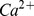
source, the expected signal-to-noise ratio in signal evoking (Type 0) experiments can be estimated and images with realistic noise can be generated. In our problem there are additional sources of fluctuations with respect to the traditional N&B method, even under stationary conditions. Besides variations in the total number of fluorescent molecules and in the number of detected photons, variations in the fraction of 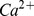-bound dye molecules also contribute to fluorescence fluctuations at each pixel. Furthermore, this fraction depends on the number of free 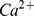 ions in the region of interest which is also a dynamic variable. Our method involves a simplification in this sense. Namely, we assume that the probability, 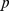, that a dye molecule be bound to 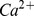 is fixed under basal (stationary) conditions and that its dynamics is governed by deterministic equations (Eqs. 14) during 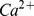
signals. Thus, in the absence of signals, the fluorescence fluctuation model of our method has five unknown parameters, the amplification factor of the detector, 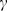, the number of emitted photons per free and 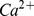-bound dye molecule that reach the detector, 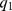
and 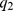, the probability that a dye molecule be bound to 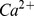, 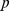, and the mean number of dye molecules that contribute to the fluorescence at a pixel, 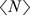. These unknown parameters reduce to four if 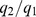 is known a priori, as assumed in this paper. When quantifying the model of our method we also assume that 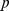 and 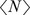 can vary from region to region of the sample (in our case, the oocyte) and between samples (*i.e.*, between oocytes). This increases the number of quantifiable parameters with respect to the N&B method. That is why our method involves the performance of more experiments than those of N&B. In one of these additional experiments the cytosolic 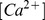 is varied which, in turn, varies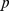. The purpose of our method is more restricted than that of the N&B technique and this simplifies the analyses. We are not trying to build a pixel-by-pixel map of 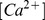. We are just interested in having a realistic quantified model of the distribution of fluorescence fluctuations.

Having a realistic fluorescence fluctuation model can be helpful to estimate the 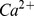
current that underlies an image. One possibility is by means of what is called a “forward approach” [26] in which the experimentally obtained image is directly compared with one that is generated via numerical simulations as those of Figs. 7A–F. Our fluorescence fluctuation model can also be useful for backward methods in which the current is inferred directly from the image [27], [28]. Even in this type of approach, once the current is inferred a numerically simulated image is generated for direct comparison with the experiments and this requires the addition of noise which is usually done in an *ad hoc* way [29], [30].

Our method can be used to identify small fluctuations that are due to 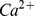 release through one or a few open channels. This is relevant for the use of optical techniques to infer single channel kinetics (the so called “optical patch clamping”) [31]. Being able to identify single channel openings is particularly important in the case of IP_3_Rs. According to a variety of experiments IP_3_Rs diffuse on the membrane of the endoplasmic reticulum [32], [33] and while some observations seem to indicate that IP_3_R clustering occurs as a consequence of stimulation with IP_3_ [15], [16], other 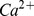 imaging experiments indicate that 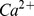
puffs occur at fixed locations in the cell [34]. Being able to tell apart spontaneous fluctuations from small changes in fluorescence due to 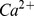 release would certainly be of help to solve this apparent paradox. Small changes in fluorescence with respect to the background also need to be detected when trying to analyze the dynamics of luminal 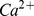
during the occurrence of localized signals as done in [35]. In [35] fluctuations due to 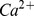 release are distinguished from spontaneous fluorescence fluctuations by means of an analysis that assumes that fluctuations follow a Poisson distribution. A model like the one constructed with our method, that quantifies the fluctuations of each specific experimental setting and that separates the background noise due to fluctuations in the number of fluorescent molecules from those in the number of detected photons that is present in all images [36] would certainly be a useful tool to identify whether a small fluctuation in luminal 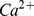 is due to 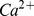
release into the cytosol or not.

### Self-consistency tests of the method

In this paper we have used our method to compare the signal detectability properties of experiments performed with two different dyes and at different concentrations. Before discussing the signal-to-noise ratios that we could estimate for the sets (***i***)–(***iii***), we do first some self-consistency tests of the method. The first test consists of determining a range of 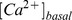 values that are compatible with the fluctuation model parameters that we estimated with the method for sets (***i***)–(***iii***). In particular, using the values of 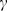 and of 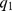 and 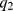 at the standard illumination power we can establish a range of possible values of 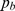 (the probability that a dye molecule be bound to 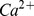 under basal conditions) that are compatible with the results derived from Type III experiments. We do it first for set (***i***). To this end we use Eq. 12 with 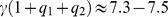 on the points of Fig. 3C with low enough fluorescence to guarantee that they correspond to basal 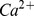 conditions. In particular, we do it for the points with 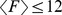 and with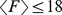. Applying Eq. 12 to these points we obtain for each of them a value of the ordinate, 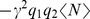. Assuming that 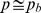 for these points we use Eq. 8 to write 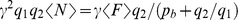. From this equality we determine a set of possible values of 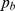. In this case we find: 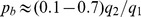. This means that, for set (***i***), the contribution of the fluorescence of the 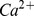-bound dye can be comparable to that of the free dye under basal conditions. Using 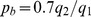, 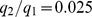 and 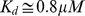 the relationship 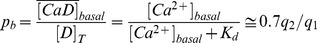 implies that 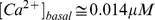 which is small but not an unreasonable value. We proceed similarly for set (***iii***). Namely, we use Eq. 12 with 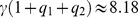 on the points of Fig. 5C with 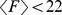 to get a range of possible values of 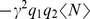. Using Eq. 8 to write 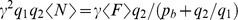 we find that 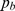 is negligible with respect to 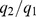. This means that the basal fluorescence is dominated by that of the free dye molecules in this case. We then conclude from our estimates that, at basal conditions almost all of the fluorescence comes from the dye free molecules in the case of Rhod-2 while the contribution of the 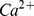-bound dye molecules is comparable to that of the free ones in the case of Fluo-4. Assuming that 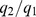 is of the order of the ratio of quantum efficiencies estimated for Rhod-2 in [24], 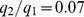, the relationship 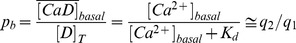 with 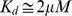 (the value provided by Invitrogen) implies that 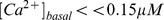
which is consistent with the estimate obtained for the case of Fluo-4 (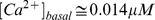). Furthermore, for the few points of set (***iii***) for which we could estimate 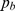 we obtained 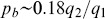 which implies 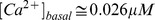, a value within the same order of magnitude as the one derived for Fluo-4.

The second test consists of comparing the range of 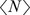 that can be inferred from the experiments of Type I for cases (***i***) and (***iii***) (Figs. 3A and 5A) with those that can be derived from the experiments of Type II (Figs. 3B and 5B). Particularly, from the experiments illustrated in Fig. 3B we can also estimate 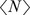 in the observed region if we assume known values of 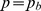 and 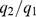. The fit of the curve in Fig. 3B, which is not very good, estimates the prefactor of the nonlinear term, 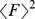, as 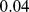. Thus, 
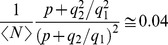
 according to Eq. 11 from which we obtain 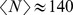 if we use 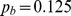 and 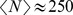 if we use 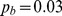. These numbers are between 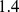 and 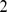 times larger than the largest one derived from Fig. 3A. The difference can be attributed to the low quality of the fit of Fig. 3B. Proceeding analogously for the case of set (***iii***), we estimate 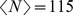 from the fit of Fig. 5B using 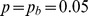. This value is within the range of 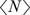 derived from Fig. 5A for the same case. The estimate becomes 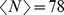 for 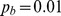 (which corresponds to 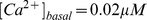). This value is smaller than the lowest estimate derived from the fit of Fig. 5A and seems to indicate that 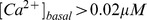 in the experiments probed in Fig. 5B.

The third test consists of comparing the range of 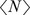 values obtained for the three sets of concentrations among themselves. From the fit to Fig. 3A for the set of concentrations (***i***) we found 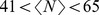 using 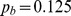 (which corresponds to 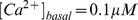 using 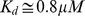) and 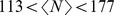 using 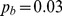 (which corresponds to 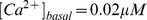). The fit of Fig. 3B did not provide reliable estimate of 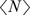. From the fit to Fig. 4A for the set of concentrations (***ii***) we obtained 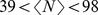 using 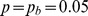 (which corresponds to 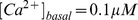 and 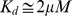) and 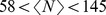 using 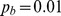 (which corresponds to 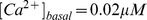)._These values are consistent with the ones derived for case (***i***) which have the same dye and EGTA concentrations._ From the fit to Fig. 5A (***iii***) we obtained 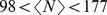 for 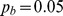
(which corresponds to 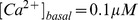) and 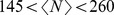 for 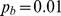 (which corresponds to 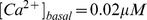). These values of 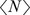 are between 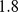 and 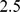 times larger in Fig. 5A than in Fig. 4A which is consistent with the fact that the dye concentration in Fig. 5A is 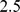 times larger than in Figs. 3A or 4A.

Summarizing, our method passed successfully a set of self-consistency tests and allowed us to estimate 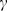, 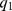 and 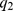 at the standard illumination power (the latter assuming known values of 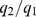) and a range of values of 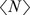 in the three examples probed in the present paper.

### Signal-to-noise ratios estimated with the method

When we applied the method to estimate the signal-to-noise ratios of the experiments illustrated in Figs. 1, it again performed very well. More specifically, the method determined that the expected signal-to-noise ratio for the conditions of Figs. 1A,1C were similar while the one of Fig. 1B was much smaller. This is consistent with the fact that we could not observe 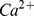 puffs under the experimental conditions of Fig. 1B, but we did observe them with the other two combinations. Thus, our method classifies the experiments of the example correctly in terms of signal detectability. Furthermore, the method can be used to determine to what extent changes in certain experimental parameters can lead to a noticeable improvement of the signal-to-noise ratio as illustrated in Fig. 8. This figure also shows how the method can be used to guarantee signal comparability between experiments performed with two different dyes when the dye used in a series of experiments needs to be changed because it is discontinued or replaced by an upgrade.

### Additional information that can be inferred with the method

Having a realistic fluctuation model, on the other hand, allows us to go beyond a mere classification and draw additional information on the imaging experimental conditions. In particular, we can investigate in more detail what factors are most important for the differential ability of the different experimental settings to detect 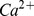
elevations. We can do so in the example analyzed in this paper. More specifically, we can explore to what extent the different kinetics of the dyes and the different EGTA concentrations are responsible for the different types of behaviors observed. To explore the effect of the different dye kinetics on the observed images, we analyzed the blurred 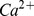-bound dye concentration obtained with the simulations. The obtained concentrations are shown in Fig. 6. A direct observation of this figure does not show significant differences in detectability among the three simulated situations that correspond to the set of concentrations (***i***) (A); (***ii***) (B) and (***iii***) (C). This coincides with a more quantitative comparison of the simulations. In particular, the ratio of the (blurred) 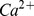-bound dye concentration at the peak of the simulated signal over its basal value is approximately the same for all three figures. We investigated to what extent these results depended on the simplified model that we used for the simulations. In particular, we added a mobile buffer and explored a wide range of these parameters always obtaining the same behavior. The results of Fig. 6 and these additional studies imply that the differences in detectability observed between the cases (***i***) or (***iii***) and those of case (***ii***) cannot be attributed to differences in the underlying 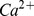
–bound dye distribution. It is only via the generation of noisy numerically simulated images by means of our quantified fluorescence fluctuation model that we can reproduce the detectability properties of the three experimental conditions probed in Fig. 1. This also shows that the ratio given by Eq. 1 is not always a faithful reporter of the increment in the 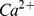 bound dye concentration during signals with respect to the same concentration at basal conditions.

The conclusion according to which the differences in detectability between the experiments of Figs, 1A and 1B cannot be accounted for by differences in the underlying
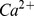
–bound dye distribution led us to ponder the role of the 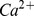-free dye fluorescence on the images. To this end, we generated noisy images combining the fluctuation model and numerical simulations but without including the contribution of the
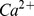
–free dye molecules (*i.e*., setting 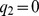). We show the results in Figs. 7D–F. Differences in detectability of the simulated signal are much less clear than in the case displayed in Figs. 7A–C. This observation is confirmed by a quantitative comparison. We show in the first column of Table 5 the signal-to-noise ratios obtained when using 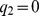. There we see that the ratio obtained for the case (***ii***) is only slightly smaller than the one for case (***i***). Furthermore, the ratio for case (***iii***) is even larger than the one for case (***i***). This does not agree with what is observed in the Type 0 experiments performed for these sets (e.g., Fig. 1). We then conclude that noise by itself cannot account for the inability of Rhod-2 to report puff occurrences when used at low concentrations. The contribution of the 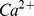
–free dye to the fluorescence is relevant for the differences in detectability observed when using Fluo-4 or Rhod-2. This is also apparent in the estimates of 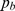 when compared with 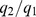 that can be derived combining the fits of Figs. 3A and 3C or 5A and 5C. Namely, from Figs. 3A and 3C we obtained 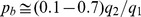
for the set of concentrations (***i***). This means that the contribution of the fluorescence of the 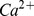-bound dye can be comparable to that of the free dye under basal conditions for this setting. Combining the results of Figs. 5A and 5C we obtained 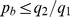 (and 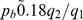 at most) for the set (***iii***). This means that basal fluorescence is mainly due to the free dye molecules for this experimental setting.

The different values of 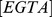 used in the experiments of Figs. 1B and 1C also play a relevant role on the better detectability properties of set (***iii***) when compared with those of set (***ii***). This is reflected in the different behaviors that the 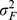
vs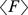 curves displayed for the two experimental settings when changing the illumination power (Figs. 4B and 5B). Namely, while this curve has a parabolic shape in Fig. 5B, it remains a straight line in Fig. 4B. This means that the nonlinear part of the 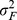 vs 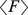 curve is not reached for the laser powers probed in the case (***ii***). According to Eq. 11 the nonlinearity of the 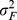
vs 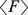 should become noticeable when 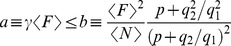. Inserting Eq. 8 into this inequality we obtain 
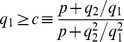
. The quantity 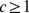 since 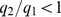. Furthermore, it is 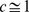 if 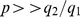 while it is 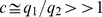 for 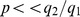. This implies that the smaller 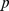
with respect to 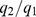, the larger 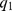 needs to be for the nonlinear term of Eq. 11 to be noticeable. Therefore, the different behavior of Figs. 4B and 5B could be attributed to a smaller value of 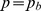 in the experiments of Fig. 4B. This, in turn, could be due to the larger amount of EGTA used in those experiments compared to the ones of Fig. 5B. This analysis together with the fact that no increment in fluorescence was observed upon 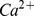 microinjection for the set of concentrations (***ii***) is an indication that EGTA is capturing 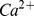 more efficiently than the dye for this experimental setting. This explains why a larger 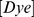 and smaller 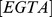 is necessary to report the occurrence of localized signals using the dye, Rhod-2, with similar detectability levels as when using Fluo-4. This analysis not only illustrates the possible applications of our method. It also indicates that, in certain cases, it is not necessary to compute the signal-to-noise ratio by means of numerical simulations to determine the inadequacy of certain combinations of dye and EGTA concentrations to observe localized 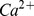
signals. In particular, it seems that we should not expect to have good signal detectability for experimental settings such that the 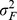
vs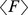 curve derived from Type II experiments.

### Final remarks

The final aim of our approach is to advance towards a more quantitative description of 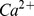
imaging experiments. It is true that the use of ratiometric dyes is more adequate if one is willing to estimate the concentration of free 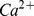. Namely, ratiometric indicators shift their peak excitation or emission wavelength upon 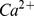 binding which allows to quantify 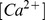 in a way that is free of the problems associated to uneven dye loading, dye leakage, photobleaching or changes in cell volume. However, these dyes are excited with wavelengths at which caged components are photolized. This makes them difficult to use in combination with caged components such as caged IP_3_. On the other hand, in order to use them for the observation of signals, it is necessary to have multi-spectral probes or to switch very rapidly between wavelengths. An alternative to the use of these dyes is to measure the mean fluorescence lifetime [37] which also serves to determine how much dye is bound to 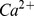 [38], [39]. It has been shown that some 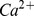
indicators, in particular, single wavelength dyes such as Oregon Green, Calcium Orange or Calcium Green, have a fluorescence lifetime that depends on 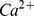. The problem with recording fluorescence decay curves is the time it takes to do it. One way to deal with this is by means of the so-called time-correlated fluorescence lifetime imaging (FLIM), but still, the method is limited by the acquisition time since enough photons need to be collected to extract reliable information and this limits its applicability [38]. Single wavelength dyes that show a 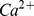-dependent lifetime could then be used combining FLIM and our method to analyze the experimental setting under basal conditions. By separating the contributions to the fluorescence from the of 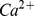-bound and the 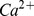-free dye molecules, FLIM would give an accurate *in situ* measurement of 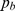 and of 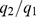. Our method would then provide estimates of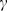, 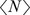
and 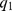 and, by means of numerical simulations, signal-to-noise ratios and numerically simulated images with realistic noise.

## Supporting Information

Text S1 **Parameter estimations from the polynomial fits.** Confidence intervals for the various fitting parameters. All estimations are done with 95% confidence level.
(PDF)
