# Supplementary material for: Fluorescence Fluctuations and Equivalence Classes of Ca 2+ Imaging Experiments
Source: PLoS One. 2014 Apr 28;9(4):e95860. doi: 10.1371/journal.pone.0095860 (PMC4002475; doi:10.1371/journal.pone.0095860)
Supplement: Text S1 — Parameter estimations from the polynomial fits. Confidence intervals for the various fitting parameters. All estimations are done with 95% confidence level. (PDF) [file pone.0095860.s001.pdf]

Supporting Information

Text S1. Parameter estimations from the polynomial fits.
Confidence intervals for the various fitting parameters.
All estimations are done with 95 % confidence level.

Experiment Type I: Fluorescence variability within a single oocyte

$\sigma_F^2 = a < F > +b$

Experiment Type II: Fluorescence variability with laser power

$\sigma_F^2 = a < F >^2 +b < F > +c$

Experiment Type III: Fluorescence variability with cytosolic calcium concentration

$\sigma_F^2 = a < F > +b$

Figure 3
(A)
$a = 4.77$ $(4.37; 5.18)$
$b = 7.05$ $(0.13; 13.97)$
(B)
$a = 0.044$ $(0.037; 0.051)$
$b = 5(fixed)$
$c = 0(fixed)$
(C)
high fluorescence
$a = 7.27$ $(6.76; 7.78)$
$b = -31.04$ $(-47.65; -14.43)$

Figure 4
(A)
$a = 5.80$ $(5.50; 6.10)$
$b = -4.77$ $(-9.93; 0.39)$
(B)
$a = 0.011$ $(0.005; 0.017)$
$b = 6.21$ $(5.81; 6.61)$
$c = -4.34$ $(-8.20; -0.47)$

Figure 5
(A)
$a = 6.23$ $(5.55; 6.92)$
$b = 1.20$ $(-22.59; 25.00)$

(B)
$a = 0.029$ $(0.027; 0.031)$
$b = 6.02$ $(5.46; 6.58)$
$c = -15.7$ $(-33.7; 3.53)$

(C)
high fluorescence
$a = 8.17$ $(7.44; 8.92)$
$b = -85$ $(-128; -42)$
